# The Role of Antioxidant Agent (N-Acetylcysteine) in Oleic Acid-Induced Acute Lung Injury in a Rat Model

**DOI:** 10.7759/cureus.29478

**Published:** 2022-09-23

**Authors:** Sudhashekhar Kumar, Priyanka Bhagat, Smriti Pandey, Ratna Pandey

**Affiliations:** 1 Department of Physiology, Institute of Medical Sciences, Banaras Hindu University, Varanasi, IND; 2 Department of Ophthalmology, Grant Medical College, Mumbai, IND

**Keywords:** ali, pulmonary edema, n-acetylcysteine, oleic acid, acute lung injury

## Abstract

Context

Reactive oxygen species (ROS) produced by inflammatory cells play a major role in mediating lung injury in sepsis or hyperoxic lung injury.

Aims

N-Acetylcysteine (NAC), an antioxidant, was examined in this research to see whether it helps prevent acute lung injury (ALI).

Materials and methods

Experiments were performed on Charles-Foster strain healthy male adult albino rats. All the animals were randomly divided into one control and two experimental groups. In control/group I, saline was administered, and cardiorespiratory parameters were recorded. Oleic acid (OA) was administered in group II to produce ALI. In group III, OA was administered to NAC-pretreated rats, and cardiorespiratory parameters were recorded to observe the effect of NAC on ALI. This study used analysis of variance (ANOVA) with two factors and a post hoc test (multiple comparisons - least significant difference (LSD) test) for statical analysis. For determining survival time, the Mantel-Cox test and Kaplan-Meier survival curves were used. A P value < 0.05 was considered significant.

Results

Respiratory arrest, pulmonary edema, and reduced partial pressure of oxygen (PaO_2_)/fraction of inspired oxygen (FiO_2_) ratio were all indications of OA-induced ALI in rats. The animals in the NAC + OA group had better respiratory and cardiac statistics than those in the OA alone group, and their survival duration was extended. However, NAC pretreatment could not protect the animals against the development of pulmonary edema.

Conclusions

These observations indicate that NAC (an antioxidant agent) protected rats against ALI in the initial phase and prolonged the survival time but failed to prevent the development of pulmonary edema.

## Introduction

One of the leading causes of death and morbidity around the globe is acute lung injury (ALI), a serious clinical concern globally [1,2]. Hypoxemia and widespread pulmonary infiltrates occur suddenly without cardiac failure, and the condition is also known as “non-cardiogenic pulmonary edema” (NCPE) [3]. A mechanically ventilated patient who developed refractory hypoxia, widespread pulmonary infiltrates, and poor compliance is termed an “acute lung injury” case [4]. Berlin’s description of the disorder states that people with ALI are more likely to have bilateral lung opacities, atelectasis or nodules in the lungs, and respiratory failure that is not fully due to cardiac-induced lung edema or volume overload. Further, the partial pressure of oxygen (PaO_2_)/fraction of inspired oxygen (FiO_2_) ratio is less than 300 mmHg but more than 200 mmHg, all features arising within <7 days from the predisposing clinical insults [5]. An oleic acid (OA)-induced animal model of ALI in rats exhibited all these characteristic features of ALI as per the American Thoracic Society (ATS) guideline [6].

The incidence of ALI varies from five to 64 people out of every 100,000 in the United States annually [6]. Critical illness of diverse etiologies, including direct or indirect lung damage, may lead to ALI [7]. The disruption of the alveolar-capillary barrier in ALI may result in increased vascular permeability and interstitial and alveolar flooding, loss of diffusion capacity, and pulmonary hypertension [2]. Type II pneumocyte injury may potentially lead to surfactant problems [8]. Experimental studies reported that in sepsis or a specific type of hyperoxic lung injury, activated neutrophils, macrophages, and stimulated pulmonary epithelial cells produce reactive oxygen species (ROS) that are very important in causing lung endothelial damage [8].

N-Acetylcysteine (NAC) is found in abundance in the lungs and serves as a precursor to reduced glutathione (GSH) [9]. NAC has natural antioxidant and anti-inflammatory properties and also has efficient mucolytic properties [9]. Reduced GSH or oxidative stress is associated with ALI and leads to poor outcomes in ALI [10]. NAC can scavenge reactive oxygen species, as it has antioxidant and anti-inflammatory properties [11]. That is why we planned to understand the role of NAC in preventing ALI in a rat model.

## Materials and methods

Animals, anesthesia, dissection, and recording

This study was conducted as per the recommendations of the ethics committee of the Institute of Medical Sciences, Banaras Hindu University, Varanasi, India, with IRB number Dean/2016 CAEC/621, ECR/526/Inst/UP/2016/RR-20. The experiments were performed on healthy adult male albino Charles-Foster rats weighing 175-225 g. The animals were randomly divided into three groups (six animals in each group). The animals were given animal food (Hindustan Lever Ltd., Mumbai, India) and water ad libitum for their nutritional requirements. The animals were kept at room temperature at 50% relative humidity in a 12-hour light-dark cycle [12]. The animals were fasted overnight and anesthetized with urethane (1.3-1.5 gm/kg body weight) [12]. Tracheal cannulation was done to keep the respiratory tract patent, jugular venous cannulation for saline/drug administration, and carotid artery cannulation for recording blood pressure (BP) via a pressure transducer (ADInstruments, Bella Vista, Australia). It was computed to determine the mean arterial pressure (MAP). A force-displacement transducer (ADInstruments, Bella Vista, Australia) was used to assess respiratory rate (RR) by securing a thread to the skin over the xiphisternum and connecting it to the transducer. Electrocardiographic (ECG) potentials for heart rate (HR) were recorded by connecting the needle electrodes in a limb lead II set up. A computerized chart recorder (Lab Chart 7, ADInstruments, Bella Vista, Australia) was used for recording.

Experimental protocol

The animals were randomly divided into three groups. After anesthesia and dissection, all the animals were stabilized for 30 minutes before starting the experimental protocol.

In group I (control) (n = 6), after initial recording, 75 µl of saline was administered through the jugular vein, and respiratory rate (RR), heart rate (HR), and blood pressure (BP) were recorded for the initial two minutes and then at the interval of 15 minutes for the next 120 minutes. This group served as a time-matched control group.

In group II (oleic acid (OA)) (n = 6), after the initial recording of RR, HR, and BP, OA (75 µl IV bolus) was injected, and the recordings were taken for the first two minutes and then at the interval of 15 minutes up to 120 minutes or till the death of animals. The dose of OA was used according to our earlier reports [13].

In group III (NAC + OA) (n = 6), after initial recordings of RR, HR, and BP, N-acetylcysteine (163 mg/kg IP) was injected to record the response of RR, HR, and BP [14]. Five minutes after NAC pretreatment, OA (75 µl IV) was administered in rats, and the response was recorded. The recordings were taken as mentioned in group II.

In all studies, blood samples were taken from the carotid artery 15 minutes after the injection of OA in a heparin-rinsed syringe. A Roche OMNI C blood gas analyzer (Roche Diagnostics GmbH, Mannheim, Germany) was used to assess the blood sample’s PaO_2_ value. As reported earlier, the PaO_2_/FiO_2_ ratio was calculated [13].

Determination of pulmonary water content

All rats had their pulmonary water content measured by physical method [12]. At the end of each experiment, the lungs were excised. The left side lung was weighed and dried to a constant weight in an electric oven at 90°C (for 48 hours), while the other side was kept in formal saline for histological examination. The differences in wet and dry lung tissue provided pulmonary water content expressed as a percentage of wet lung tissue.

Histology of the lung

The lungs preserved in formal saline were subjected to standard histological protocol and stained with hematoxylin and eosin for microscopic examination.

Drugs and solutions

The drugs and solutions used were urethane from Sigma-Aldrich (St. Louis, USA), OA from HiMedia Laboratories (Mumbai, India), and NAC from Samarth Life Sciences (Mumbai, India).

Statistical analysis

The Statistical Package for the Social Sciences (SPSS) software version 16.0 (SPSS Inc., Chicago, USA) and SigmaPlot software version 10.0 (Systat Software, San Jose, CA, USA) were used for data analysis and graphical presentation. Data were presented as mean ± standard error of the mean (SEM). The time-response relation among the groups was performed using a two-way analysis of variance (ANOVA) followed by a post hoc test (multiple comparisons - least significant difference (LSD) test) to find a pair-wise difference in mean values. Further, comparisons between corresponding times and pulmonary water in these groups were performed by one-way ANOVA followed by a post hoc test (multiple comparisons - Bonferroni test) to find differences in mean values. Kaplan-Meier survival curves and the log-rank (Mantel-Cox) test were used to compare the survival time between different groups. A P value < 0.05 was considered significant.

## Results

Saline administration produced no significant change in the respiratory and cardiac parameters

**Figure 1:**
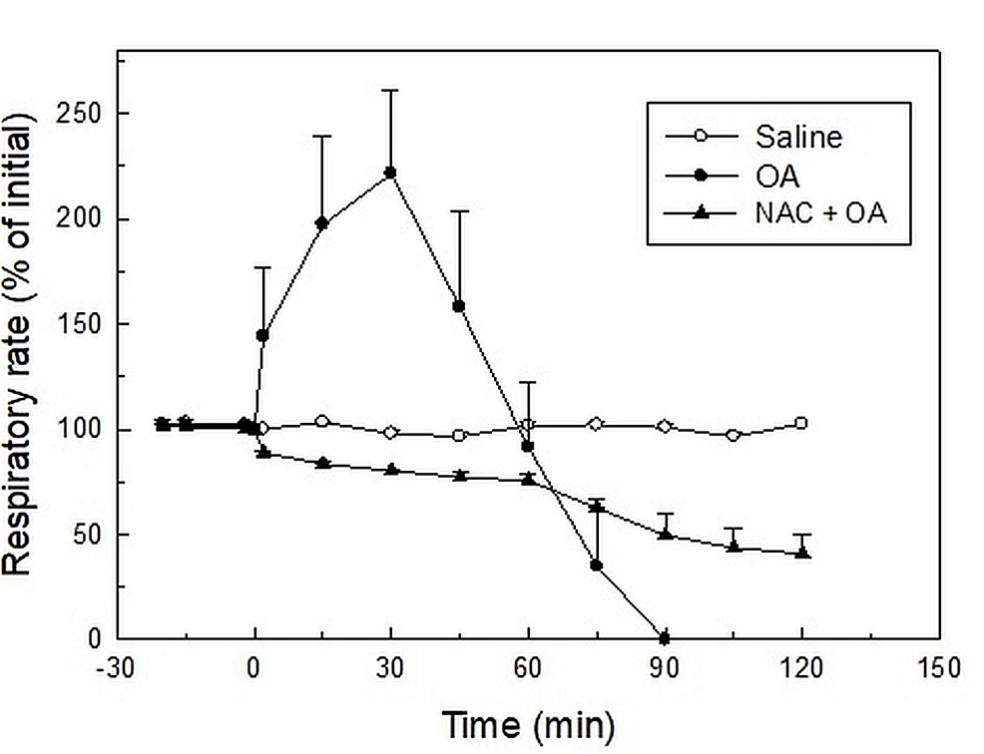
Effect of OA (75 µl) on respiratory rate (% of initial) in OA and NAC-pretreated (NAC + OA) group. NAC was injected five minutes prior to the injection of OA. The respiratory rate at 0 minutes (time of injection of OA) was taken as 100%. Mean ± SEM values were obtained from six different experiments in control, OA, and NAC + OA groups. Changes in the NAC + OA group were significantly different from the OA group (P < 0.05, two-way ANOVA). Saline: control group; OA group: oleic acid-treated group; NAC + OA: N-acetylcysteine + oleic acid-treated group; SEM: standard error of the mean; min: minutes

Rats administered with saline (as a control) had an RR mean ± SEM of 79.67 ± 1.51 breaths per minute. For the duration of the data collection, there was no discernible change in RR (Figure 1).

**Figure 2:**
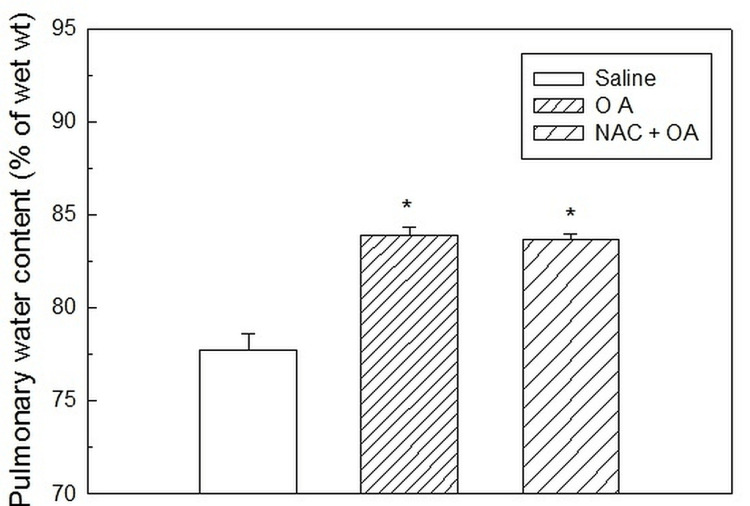
Pulmonary water content (% of wet lung tissue) in OA-treated, NAC-pretreated (NAC + OA), and saline groups. The values are expressed as mean ± SEM and are obtained from six different experiments in each group. The asterisk (*) indicates a significant difference from the control group (P < 0.05). Saline: control group; OA group: oleic acid-treated group; NAC + OA: N-acetylcysteine + oleic acid-treated group; SEM: standard error of the mean

In the control group, the pulmonary water content was 77.74 ± 0.83% (Figure 2, Table 1).

**Table 1:** Qualitative assessment of various parameters in the OA group and NAC + OA group as compared to the saline (control) group. The “+,” “++,” and “+++” and “-” signs depict comparative increases or decreases in the parameters, respectively. OA: oleic acid; NAC: N-acetylcysteine; RR: respiratory rate; PE: pulmonary edema; RBC: red blood cells; MAP: mean arterial pressure; PaO_2_: partial pressure of oxygen; FiO_2_: fraction of inspired oxygen

Parameters	Saline (control) group	OA group	NAC + OA group
RR	Continued for >120 minutes	Stopped by 90 minutes	Continued for >120 minutes
PE	-	+++	+++
Histopathological alteration as compared to saline (control) group			
Alveolar damage	-	+++	+
Infiltration of inflammatory cells and RBC	-	+++	+
Exudation	-	+++	+
HR	Continued for >120 minutes	Stopped by 90 minutes	Continued for >120 minutes
MAP	Continued for >120 minutes	Stopped by 90 minutes	Continued for >120 minutes
PaO_2_/FiO_2 _ratio	454.67 ± 3.87 mmHg	250.40 ± 9.03 mmHg	360.10 ± 5.16 mmHg
Survival time	>120 minutes	<90 minutes	>120 minutes

**Figure 3:**
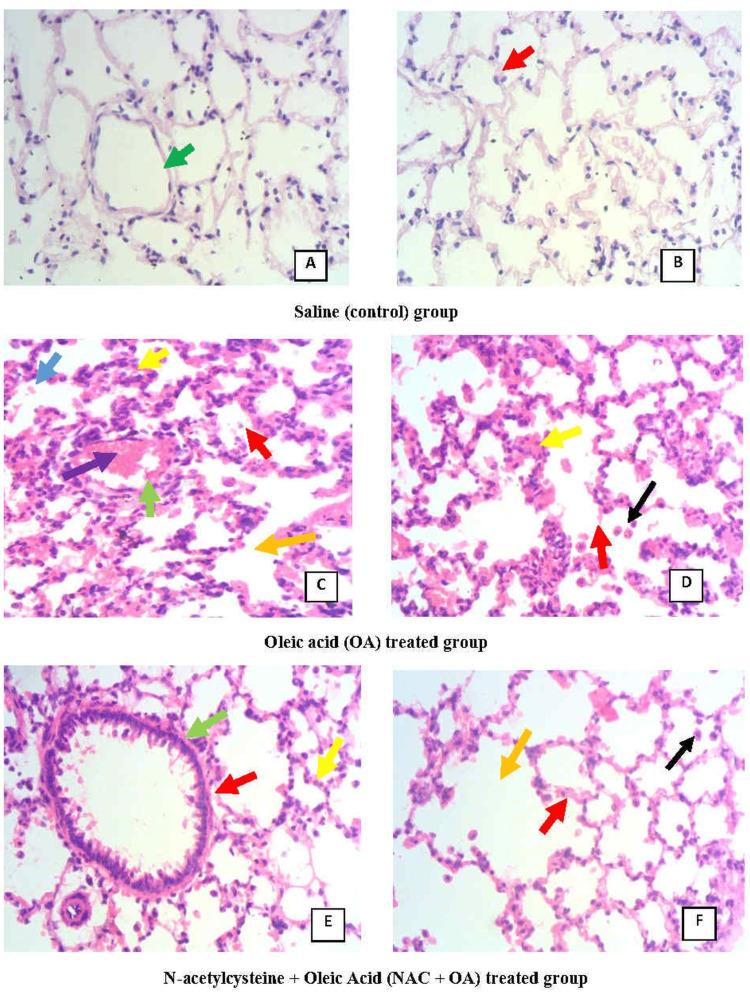
Photomicrograph of rat lung in saline (A and B), OA (C and D), and NAC + OA (E and F) groups. “Red arrow” depicts simple squamous epithelium of alveoli. “Green arrow” depicts the endothelial lining of blood vessels. “Yellow arrow” depicts the thickening of the interalveolar septa. “Black arrow” depicts infiltrated cells. “Blue arrow” depicts RBC. “Purple arrow” depicts intravascular congestion. “Orange arrow” depicts merged alveolar cavity. Magnification: 400× Saline: control group; OA group: oleic acid-treated group; NAC + OA: N-acetylcysteine + oleic acid-treated group; RBC: red blood cells

The mean ± SEM of the PaO_2_/FiO_2_ ratio was 454.67 ± 3.87 mmHg (Table 1). Histological investigation of rat lung parenchyma revealed normal alveoli. Alveoli were lined by simple squamous epithelium. The interalveolar septum was shared by adjacent alveoli (Figure 3).

**Figure 4:**
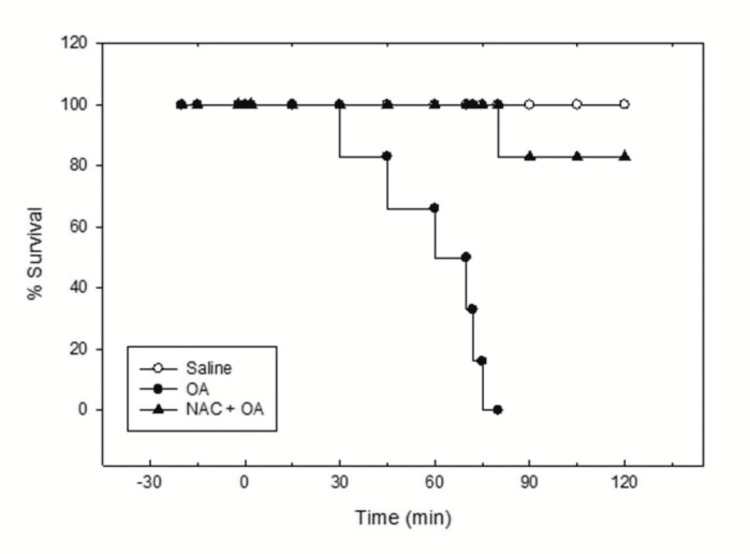
Kaplan-Meier survival plot for saline, OA, and NAC + OA groups. The survival time for the NAC + OA group is significantly greater than the OA group (log-rank (Mantel-Cox) test). Saline: control group; OA group: oleic acid-treated group; NAC + OA: N-acetylcysteine + oleic acid-treated group; min: minutes

The heart rate and mean arterial pressure in this group of rats were 301.67 ± 5.25 beats per minute and 72.00 ± 1.15 mmHg, respectively. Throughout the trial, their levels did not vary much (Table 1). The animals in the control group were healthy and alive for the whole measurement time of 120 minutes (Figure 4, Table 1).

**Figure 5:**
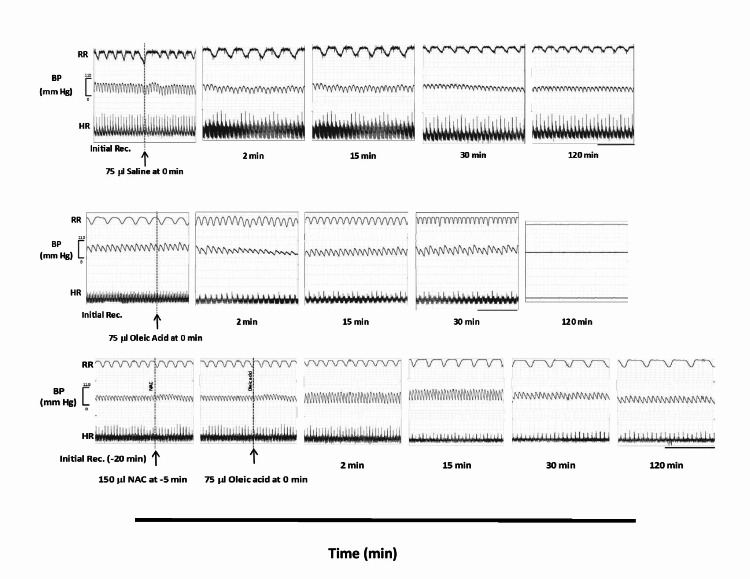
Representative prototypes of original recordings showing the effects of OA 75 µl on respiratory frequency (RR), BP, and ECG (HR) from a single experiment in the OA (middle panel) and NAC + OA (lower panel) groups. The upper panel shows the control recording (saline). In each panel, the initial part shows the control recordings. The dotted line indicates the injection of oleic acid. The horizontal line is the time calibration for respiration and ECG (two seconds). The vertical calibration and the numerical associated with BP indicates the value in mmHg. OA: oleic acid; NAC + OA: N-acetylcysteine + oleic acid; RR: respiratory rate; BP: blood pressure; HR: heart rate; ECG: electrocardiography; min: minutes

Figure 5 (upper panel) shows representative prototypes of original recordings.

Oleic acid produced acute lung injury in rats

In the OA group, the initial value of RR was 78.02 ± 2.13 breaths per minute. After 30 minutes of OA (75 µl IV bolus) administration, RR increased twice the initial value. Subsequently, the RR decreased progressively, and the respiration stopped by 90 minutes (Figure 1). This group’s pulmonary water content was significantly higher (83.91 ± 0.44%; P < 0.05) (Figure 2, Table 1) as compared to the control group (77.74 ± 0.43%). The lung histology revealed lung parenchymal destruction with collapsed alveoli. In certain regions, the lung architecture was completely destroyed, and the alveolar space significantly decreased. The thickening of the interalveolar septa, a sign of interstitial edema, is caused by leucocyte infiltration. The histology slides also noted endothelial cell damage and intravascular congestion (Figure 3). The PaO_2_/FiO_2_ ratio value (250.40 ± 9.03 mmHg; P < 0.05) (Table 1) was significantly lower compared to the control group. This set of rat’s HR and MAP were 280 ± 12.65 beats per minute and 83.83 ± 6.59 mmHg, respectively, at their baseline. The administration of OA to the animals led to a rapid drop in HR (43%) and MAP (about 45%), followed by some increase by 15 minutes, and then a steady decline after 30 minutes until the animals died (Table 1). Compared to the control group, this group’s mean ± SEM survival time (69.5 ± 3.55 minutes) was considerably less (Table 1, Figure 4, Kaplan-Meier survival curve and log-rank (Mantel-Cox) test). These original recordings are shown in Figure 5 (middle panel).

N-Acetylcysteine pretreatment protected rats against oleic acid-induced injury

The RR, HR, and MAP were not significantly altered by N-acetylcysteine pretreatment. The administration of OA (75 µl) in NAC-pretreated rats did not produce tachypnea as observed in the OA group. OA treatment reduced the RR in NAC-pretreated rats, but the RR was maintained at a reasonable level (75% of the initial) for up to 60 minutes. The RR continued to decline after 60 minutes, but even at 120 minutes, it was 59% of the initial value (Figure 1). However, compared to the control group, the pulmonary water content of NAC-pretreated rats (83.65 ± 0.29%) was significantly higher (P < 0.05; Figure 2, Table 1). The pulmonary water content of this group was comparable to that of the OA-treated group. Histological examination of lungs showed multifocal and heterogenous destruction of the lung parenchyma and the merging of several alveoli into one big cavity. However, the injury was less severe as compared to the OA-treated group. Interstitial edema, endothelial injury or intravascular congestion, and inflammatory cells were less than in the OA group (Figure 3). The PaO_2_/FiO_2_ ratio in the NAC-pretreated group was 360.10 ± 5.16 mmHg. The value was significantly greater than that in the OA-treated group (250.40 ± 9.03 mmHg; P < 0.05) but less than that in the control group (454.67 ± 3.87 mmHg) (Table 1). The administration of OA did not produce a profound fall in HR and MAP as observed in the OA group and was maintained at a good level (around 78% of initial) until 75 minutes. After this, there was a fall in HR and MAP, and by 120 minutes, HR and MAP were about 50% and 36%, respectively, of the initial value (Table 1). The Kaplan-Meier survival plot showed that the survival time in this group (115 ± 5 minutes, log-rank (Mantel-Cox) test; Figure 4, Table 1) was considerably longer than in the OA group. Figure 5 (lower panel) shows representative prototypes of original recordings.

## Discussion

ALI is characterized by hypoxemia, inflammation, lung parenchymal damage, and pulmonary edema development [15]. For experimental studies, oleic acid (OA) administration induced ALI in rats as per ATS guidelines [6,13]. OA administration induces ALI in these rats as it produces tachypnea in the early stage, indicating hypoxemia. Histological examination also shows inflammatory cells and lung parenchymal damage in these rats. OA also produced pulmonary edema in these rats compared to the control group. N-Acetylcysteine (NAC) pretreatment prevented initial rapid deterioration in respiratory and cardiac parameters in the ALI animal model induced by oleic acid administration in rats and improved the overall survival time. However, it failed to protect the rats against developing pulmonary edema and lethality. A study on a mechanically ventilated rat with lipopolysaccharide-induced ALI reported that a high dose of NAC reduced oxidative damage and inflammatory cell in the histology of the lungs [14]. Other studies also reported that NAC treatment protected against lipopolysaccharide-induced ALI [16].

Acute lung injury is thought to be caused by excess production of reactive oxygen species (ROS) due to oxidative stress, the oxidant damage they induce, and decreased antioxidant levels [5,8]. Previous reports show that oxidative stress is related to poor results in ALI [17]. The effective removal of ROS by antioxidants may be beneficial in the ALI treatment strategy reported in clinical studies [18]. N-Acetylcysteine (NAC) is a common antioxidant and anti-inflammatory drug [19]. Meta-analysis shows that the administration of NAC did not reduce short-term mortality, 30-day mortality, or the PaO_2_/FiO_2_ ratio, although it reduced the duration of ICU stay [17]. Few clinical studies of coronavirus disease (COVID-19)-induced ALI also reported that the use of NAC was beneficial, along with another treatment strategy [20]. The use of NAC shows suppressed virus replication, reduced inflammation, and improved outcomes in these patients [20-22]. However, the benefit of NAC therapy is not consistent among these studies.

N-Acetylcysteine is a precursor of glutathione (GSH), an antioxidant at significant levels in the lungs [9]. ALI patients have a deficiency of GSH in lung lavage, indicating a reduced level of GSH during an acute episode of ALI [15]. NAC promotes GSH production and has natural antioxidant properties [9]. NAC can scavenge reactive oxygen species, including hydrogen peroxide, superoxide anion, and hypochlorous acid. NAC reduces ROS-induced nuclear factor kappa B (NF-κB) activation, which plays an important role in the inflammatory cascade and suppresses the release of inflammatory cytokines [21]. In this study, the administration of NAC reduced inflammatory cells and endothelial damage to some extent, as shown in lung histology. Further, NAC has an effective mucolytic property, which loosens thick mucus in the lung and improves lung function, as indicated by improvements in early respiratory parameters. However, although NAC positively impacted lung damage in the early stages, it failed to prevent the development of pulmonary edema.

The study limits testing only one drug with a single dose, but various other agents with multiple doses can also be checked.

## Conclusions

In conclusion, oxidative injury seems to play a vital role in ALI, and an antioxidant (N-acetylcysteine) is beneficial in the early phase of ALI and prolongs the survival time but is unable to prevent the development of pulmonary edema. More experiments are required to delineate the mechanism causing deterioration in the later phase of ALI.
